# Molecular characterization and phylogenetic analysis of the first *Corynebacterium rouxii* strains isolated in Brazil: a recent member of *Corynebacterium diphtheriae* complex

**DOI:** 10.1186/s12863-023-01167-w

**Published:** 2023-11-08

**Authors:** Juliana Nunes Ramos, Max Roberto Batista Araújo, Paulo Victor Pereira Baio, Lincoln Oliveira Sant’Anna, João Flávio Carneiro Veras, Érica Miranda Damásio Vieira, Mireille Ângela Bernardes Sousa, Carlos Henrique Camargo, Cláudio Tavares Sacchi, Karoline Rodrigues Campos, Marlon Benedito Nascimento Santos, Sérgio Bokermann, Luige Biciati Alvim, Louisy Sanches dos Santos, Ana Luiza de Mattos-Guaraldi, Verônica Viana Vieira

**Affiliations:** 1 Laboratory of Diphtheria and Corynebacteria of Clinical Relevance, Department of Microbiology, Immunology and Parasitology, Rio de Janeiro State University, Rio de Janeiro, Brazil; 2 Operational Technical Nucleus, Hermes Pardini Institute, Microbiology, Belo Horizonte, Brazil; 3 Army Chemical and Pharmaceutical Laboratory, Ministry of Defense, Brasília, Brazil; 4 Laboratório Interdisciplinar de Pesquisas Médicas (LIPMED) - Instituto Oswaldo Cruz - Fundação Oswaldo Cruz (Fiocruz), Av. Brasil, 4365. Pavilhão Cardoso Fontes, 1°. andar, sala 17. Manguinhos, Rio de Janeiro, CEP:21040-900 Brazil; 5 Center of Bacteriology, Adolfo Lutz Institute, Secretary of Health of the State of São Paulo, São Paulo, Brazil; 6 Strategic Laboratory, Adolfo Lutz Institute, Secretary of Health of the State of São Paulo, São Paulo, Brazil; 7 Operational Technical Nucleus, Research and Development, Hermes Pardini Institute, Belo Horizonte, Brazil

**Keywords:** *Corynebacterium rouxii*, *Corynebacterium diphtheriae* complex, Non-toxigenic, Virulence factors, Resistance genes, CRISPR-Cas system

## Abstract

**Background:**

*Corynebacterium diphtheriae* complex was formed by the species *C. diphtheriae*, *Corynebacterium ulcerans* and *Corynebacterium pseudotuberculosis* in the recent past. In addition to *C. diphtheriae*, *C. ulcerans* and *C. pseudotuberculosis* species can carry the *tox* gene, which encodes diphtheria toxin. Currently, three new species have been included in the complex: *Corynebacterium rouxii, Corynebacterium silvaticum*, and *Corynebacterium belfantii*. *C. rouxii* is derived from the ancient Belfanti biovar of *C. diptheriae.* We provide the complete genome sequences of two non-toxigenic strains *C. rouxii* isolated from a cat with a purulent infection in Brazil. The taxonomic status and sequence type, as well as the presence of resistance and virulence genes, and CRISPR-Cas system were additionally defined.

**Results:**

The genomes showed an average size of 2.4 Mb and 53.2% GC content, similar to the type strain of the species deposited in Genbank/NCBI. Strains were identified as *C. rouxii* by the rMLST database, with 95% identity. ANI and DDH *in silico* were consistent with values above the proposed cut-off points for species limit, corroborating the identification of the strains as *C. rouxii*. MLST analyses revealed a new ST, which differs from ST-537 only by the *fusA* allele. No horizontal transfer resistance gene was predicted in both genomes and no mutation was detected in the constitutive genes *gyrA* and *rpoB.* Some mutations were found in the seven penicillin-binding proteins (PBPs) detected. The *tox* gene was not found, but its regulatory gene *dtxR* was present. Among the predicted virulence genes are those involved in iron uptake and adherence, in addition to the DIP0733 protein involved in epithelial cell adhesion and invasion. The CRISPR-Cas type I-E system was detected in both genomes, with 16 spacer sequences each. Of them, half are unknown according to the databases used, indicating that there is an unexplored reservoir of corynebacteriophages and plasmids.

**Conclusions:**

This is the first genomic study of *C. rouxii* reported in Brazil. Here we performed taxonomic analysis and the prediction of virulence factors. The genomic analyses performed in this study may help to understand the potential pathogenesis of non-toxigenic *C. rouxii* strains.

## Background

The genus *Corynebacterium* currently includes approximately 140 species [1]. The best-known species of the genus is the human pathogen *Corynebacterium diphtheriae*, the etiologic agent of diphtheria, a potentially fatal infection that affects the respiratory tract and occasionally skin [2]. Diphtheria toxin (DT) is the main virulence factor of *C. diphtheriae* codified by the *tox* gene. This gene is carried by corynebacteriophages which can lysogenize *C. diphtheriae* species, leading to the conversion of an atoxigenic to a toxigenic isolate [3]. It has already been described, however, that two other species of the genus, *Corynebacterium ulcerans* and *Corynebacterium pseudotuberculosis* can also be lysogenized, and therefore can also cause diphtheria. These two species are predominantly isolated from animals [4].

In the recent past, the species *C. diphtheriae*, *C. ulcerans* and *C. pseudotuberculosis* formed the *C. diphtheriae* complex with potential to cause different infectious processes in humans and animals [5]. The species *C. ulcerans* and *C. pseudotuberculosis* can also carry the *tox* gene. Recently, diphtheria caused by *C. ulcerans* is more common in industrialized countries, being increasingly recognized as an emerging pathogen [6]. *C. pseudotuberculosis* is the etiologic agent of caseous lymphadenitis in small ruminants, such as sheep and goats [7]. Although infections rarely affect humans, cases of lymphadenitis related to occupational infections have been reported, affecting rural workers who have frequent contact with the herd or who work in slaughterhouse [8].

Currently, the *C. diptheriae* complex is formed by three more recently described species: *Corynebacterium belfantii*, *Corynebacterium silvaticum* and *Corynebacterium rouxii*. *C. silvaticum* is a novel species of the nontoxigenic *tox-*gene-bearing (NTTB) strains, firstly isolated from lymph nodes of wild boars with severe lesions due to caseous lymphadenitis [2]. The species *C. belfantii* is derived from the ancient Belfanti biovar of *C. diptheriae*. Atypical strains of the same biovar gave origin to another species, *C. rouxii* [5].

*C. rouxii* strains were isolated between 2011 and 2017 in France from human infections of the skin or peritoneum and one isolated from a dog and described by Badell and collaborators in 2020, based on biochemical and genomic taxonomy [9]. Although biochemically similar to *C. belfantii*, *C. rouxii* strains are negative for maltose fermentation. The average nucleotide identity (ANI) between *C. rouxii* isolates and *C. diphtheriae* and *C. belfantii* was 92.4% and 91.4%, respectively, values lower than the currently recommended threshold values for species limit [10, 11].

In this study, two non-toxigenic strains of *C. rouxii* were collected from a cat with a purulent infection. This is the first genomic study of *C. rouxii* reported in Brazil. We defined the taxonomic status and sequence type, as well as the presence of resistance and virulence genes, and the CRISPR-Cas system. These results may help to understand the potential pathogenesis of non-toxigenic strains of the species of *C. diphtheriae* complex.

## Results

### General features of genome sequencing

The average genome size is approximately 2.4 Mb. The genomes had predicted G + C contents of 53.2%. The assemblies of strains 70,862 and 70,863, as well N50, number of CDS, RNA and median coverage are shown in the Table 1.

**Table 1:** General features of genome sequences of two strains of *Corynebacterium rouxii*

Strains	Contigs	Genome size	N50	Median coverage	GC%	Number of CDS	Number of RNA
70,862	29	2.399,341	242.682	54x	53.2	2447	59
70,863	24	2.380.082	239.896	112x	53.2	2397	58

### Identification of species and genomic taxonomy

**Fig. 1:**
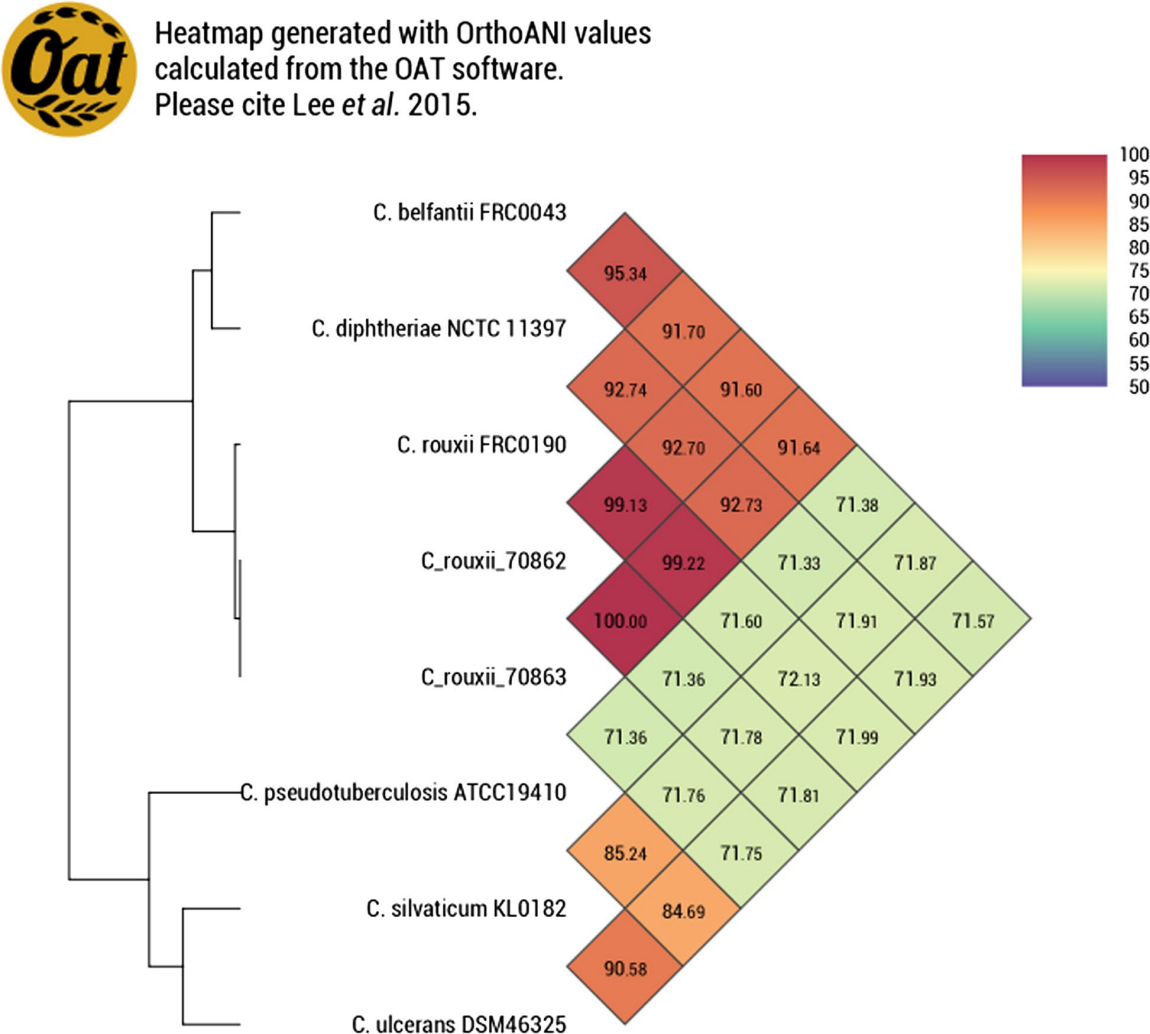
Heatmap generated by OrthoANI Tool version 0.93.1 [12] indicating high ANI values (above 99%) between the *Corynebacterium rouxii* isolates and FRC0190 type strain

According to the rMLST database, the strains were identified as *C. rouxii* with 95% identity. The genomic sequencing results showed ANI values of 99.13% and 99.22% to *C. rouxii* 70,862 and 70,863 strains, respectively, when compared to *C. rouxii* FRC0190^T^ (Fig. 1). The value of 100% was found when strain 70,862 was compared with strain 70,863 (Fig. 1). In relation to DDH *in silico* results, as expected, comparisons with the *C. rouxii* FRC0190^T^ revealed values of 92.70% and 93.10% (Table 2).

**Table 2:** DDH in silico results obtained by GGDC calculator of *Corynebacterium rouxii* strains compared to 6 closely related taxa

Strains	70,862	70,863	*Corynebacterium rouxii* FRC0190^T^	*Corynebacterium belfantii* FRC0043^T^	*Corynebacterium diphtheriae* NCTC11397^T^	*Corynebacterium pseudotuberculosis* ATCC19410^T^	*Corynebacterium silvaticum* KL0182^T^	*Corynebacterium ulcerans* DSM46325^T^
**70,862**	-	100%	92.70%	44.90%	48.60%	21.50%	21.80%	22.10%
**70,863**	100%	-	93.10%	44.90%	48.60%	21.50%	21.80%	22.20%

#### MLST characterization

The strains were classified as belonging to the new ST-899. The allelic profile is 37-25-91-26-61-21-17 for *atpA, dnaE, dnaK, fusA, leuA, odhA* and *rpoB* genes, respectively.

#### Phylogenetic analysis

**Fig. 2:**
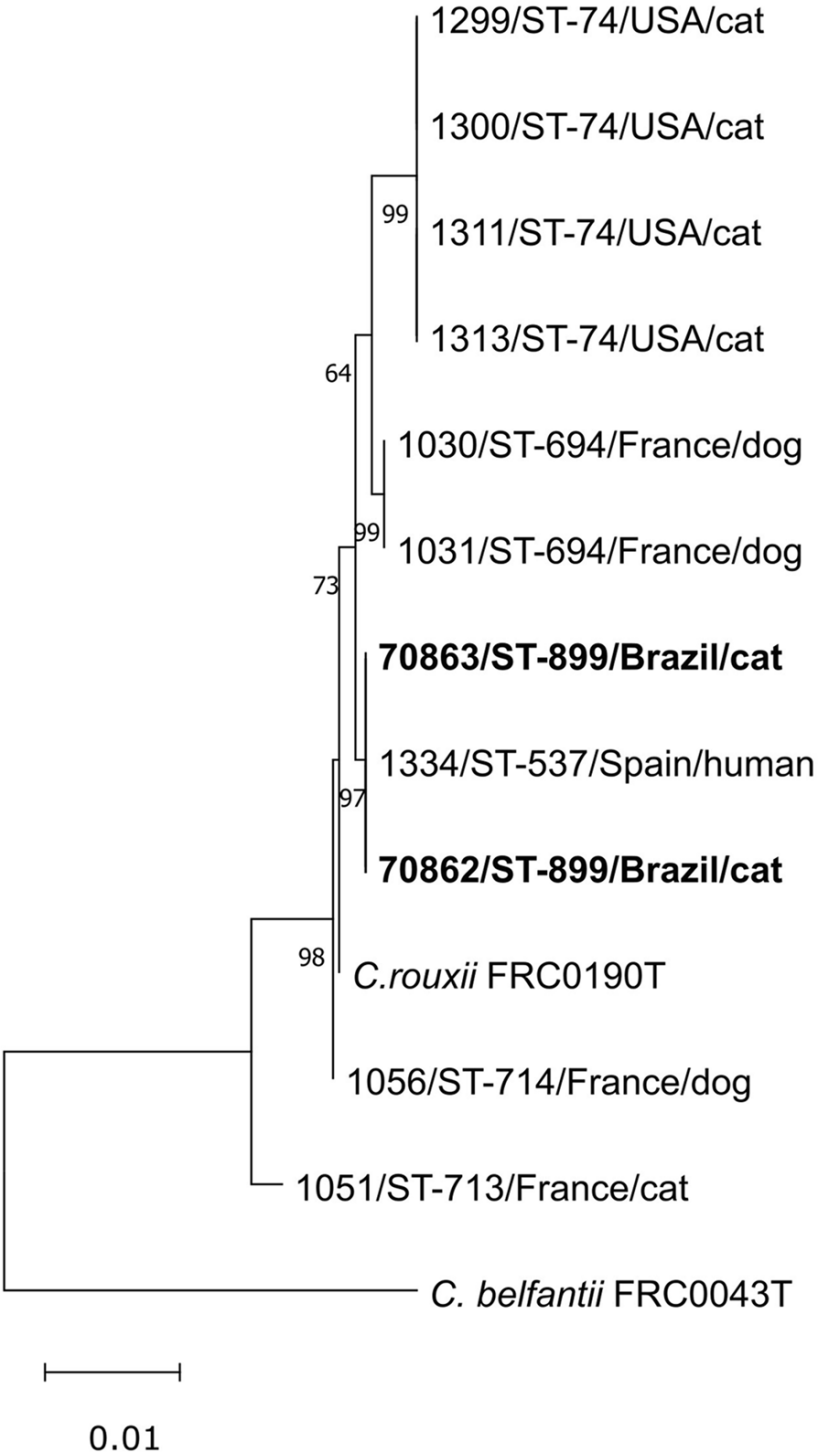
Concatenated phylogenetic tree based on the sequences of 7 housekeeping genes (2544 positions) used in the MLST scheme of *Corynebacterium diphtheriae*. The distance was inferred based on the Maximum-Likelihood method (Kimura-2 parameters). Bootstrap values with 1000 replicates. The scale bar indicates a 0.01% divergence. Highlighted (bold) are the strains of this study. The type strain of *Corynebacterium belfantii* FRC0043 was used as an outgroup

*C. rouxii* strains 70,862 and 70,863 classifieds as ST-899 clustered with the only strain belonging to the ST-537, isolated from human, in Spain (Fig. 2). Both STs diverge only in one allele. One strain (FRC0810) from the MLST database was excluded because they did not present one of the 7 alleles.

#### Prediction of virulence factors

Pilus genes clusters such as *spaA* and *spaD* involved in adherence were also found in both genomes (Table 3). Furthermore, *sapD* gene, encoding surface-anchored pilus protein, and genes involved in ABC transporter of iron uptake were also found in both genomes. Gene clusters involved in iron uptake were found in addition to sigma A factor of *Mycobacterium tuberculosis*. The *tox* gene was not found, but the *dtxR* regulator gene was found in both strains.

**Table 3:** Virulence factors predicted in Brazilian strains of *Corynebacterium rouxii*

VFclass	Virulence factors	Related genes	strains	
			70,862	70,863
Adherence	SpaA-type pili	*spaA*	+	+
		*spaB*	+	+
		*spaC*	+	+
		*srtA*	+	+
	SpaD-type pili	*spaD*	+	+
		*srtB*	+	+
		*srtC*	+	+
	Surface-anchored pilus proteins	*sapD*	+	+
Iron uptake	ABC transporter	*fagA*	+	+
		*fagB*	+	+
		*fagC*	+	+
		*fagD*	+	+
	ABC-type heme transporter	*hmuT*	+	+
		*hmuU*	+	+
		*hmuV*	+	+
	Ciu iron uptake and siderophore biosynthesis system	*ciuA*	+	+
		*ciuB*	+	+
		*ciuC*	+	+
	Siderophore-dependent iron uptake system	*irp6A*	+	+
		*irp6B*	+	+
		*irp6C*	+	+
Regulation	Diphtheria toxin repressor DtxR	*dtxR*	+	+
	Sigma A (*Mycobacterium*)	*sigA/rpoV*	+	+

#### CRISPR-Cas system

**Fig. 3:**
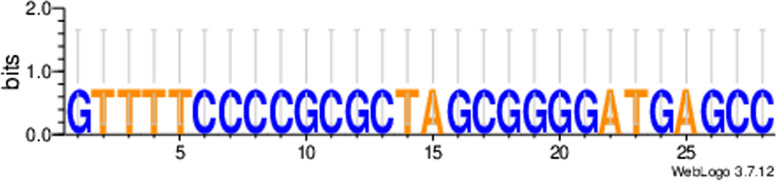
Conservation of the direct repeats in type I-E of CRISPR of *Corynebacterium rouxii* strains. The sequence logo was created by WebLogo 3.7.12 [14]. The height of the letters shows the relative frequency of the corresponding nucleotide at that position

A total of two CRISPR-Cas systems were found, one in each strain. Both systems showed a high precision score (evidence level = 4; as high as possible) based on the parameters used by the CRISPRFinder database, which assigns evidence levels from 1 to 4 for spacer repetition and similarity [13]. The CRISPRFinder server identified the type I-E. A total of 16 spacer sequences were found in CRISPR arrays of both strains. Using the CRISPR-Cas + + database, only 1 spacer returned a match for CRISPR-Cas I-E *C. diphtheriae* bv. mitis PC0646 with 100% similarity. The CRISPRTarget server identified half of the spacers (8/16) with values above 80% identity with a match for *C. diphtheriae* (*n* = 4), *C. ulcerans* (*n* = 2), *Marinobacter nauticus* (*n* = 1) and *Syntrophomonas wolfei* (*n* = 1). Eight spacers are unknown according to the database used. Direct repeat consensus sequences in both systems were identical and their conservation is shown in Fig. 3.

#### Antimicrobial resistance genes

No antimicrobial resistance genes (ARGs) were detected in both genomes, as well as mutations in the *gyrA* and *rpoB* genes, responsible for resistance to quinolones and rifampicin in corynebacteria [15, 16]. Mutations were found within 6 chromosomal penicillin-binding proteins (PBPs) coding sequences comparing to the type strain FRC0190^T^: PBP1a, PBP1b, PBP2a, PBP2b, PBP2c, and PBP4b (Table 4). No mutations were found in the *PBP4* gene.

**Table 4:** Description of the mutations found in the penicillin-binding proteins

Strains	Penicillin-binding proteins (PBPs)						
	PBP1a	PBP1b	PBP2a	PBP2b	PBP2c	PBP4	PBP4b
70,862 and 70,863	416: S➜P 430: S ➜G 669: V ➜A 699: A ➜V	699: I ➜V	181: S ➜A	69: V ➜A	196: H ➜Y 260: P ➜L	no mutations	34: V ➜A 362: T ➜S

The numbers before the amino acid abbreviations indicate the site where the mutations occurred

Amino acids abbreviations: *S* serine, *P* proline, *G* glycine, *V* valine, *A* alanine, *I* isoleucine, *H* histidine, *Y* tyrosine, *L* leucine, *T* threonine

Gene location in the type strain FRC0190 with the respective locus_tag in parentheses: PBP1a (CIP100161_RS11445); PBP1b (CIP100161_RS01510); PBP2a (CIP100161_RS00415); PBP2b (CIP100161_RS08085); PBP2c (CIP100161_RS07615); PBP4 (CIP100161_RS10180); and PBP4b (CIP100161_RS03210)

## Discussion

Non-toxigenic strains of *C. rouxii* were collected in this study and identified according to the complete genome sequences. *C. rouxii* strains have been isolated from cats, domestic dogs, and free-roaming red fox. Cases of infections with severe otitis in cats and purulent orbital cellulitis, otitis, rhinitis and ulcerative skin lesions in dogs have been reported [5, 9, 17].

The confirmation of species identification of the 2 strains was performed using taxonomic analysis, such as ANI and DDH *in silico*, in addition to identification by the rMLST database, a methodology based on 53 genes encoding the bacterial ribosome protein subunits (*rps* genes) [18]. The values obtained for ANI were consistent and above the proposed cut-off point for the species limit (95 ~ 96%) [11, 19]. The strains in this study showed DDH values above the limit (70%) for species definition [20].

MLST analysis revealed a new sequence type. ST-899 has been attributed to the two strains and is closer to ST-537, isolated from a bone biopsy of *Homo sapiens* in Spain, according to the MLST database. To date, there are only 10 strains of *C. rouxii* deposited in the MLST database, from France, the United States of America and Spain, with the following STs assigned: 74, 537, 694, 713 and 714. In the phylogenetic analysis using the MLST genes performed with the two Brazilian *C. rouxii* strains of this study plus nine *C. rouxii* strains available at the MLST database, it was possible to verify the distribution of Brazilian strains in the same clade as the representative of ST-537 (Fig. 2). ST-899 and ST-537 differ only in the *fusA* allele, 26 and 54, respectively, according to the MLST database.

Beta-lactam antimicrobials are antibacterial agents that inhibit bacterial cell wall synthesis as a result of their strong covalent binding to PBPs, which catalyze the last reaction of cell wall formation in Gram-positive and Gram-negative bacteria [21]. PBPs are target binding sites for β-lactams [22]. Seven PBPs reported in *C. diphtheriae* species were found in Brazilian *C. rouxii* genomes. Of these, six present amino acid mutations compared to the *C. rouxii* FRC0190^T^ (Table 4), but the correlation between mutations and the phenotype of resistance or susceptibility to antimicrobials will be published in a subsequent study.

Some studies have shown an increase in the rate of antimicrobial resistance among *Corynebacterium* species [15, 16, 23], but horizontal transfer resistance genes were not predicted in both genomes, according to the Resfinder database. Furthermore, there are no mutations in housekeeping genes, such as *gyrA* and *rpoB*, which lead to resistance to quinolones and rifampicin, respectively [15, 16].

Potential virulence factors were also predicted in the genomes (Table 3). The diphtheria toxin was not found in the strains, but its regulatory gene *dtxR* was present [24]. Among the virulence factors, genes involved in regulation of diphtheria toxin, iron uptake and adherence were found in these non-toxigenic strains. The surface-anchored pilus protein *sapD* involved in the adherence was found. Sigma A factor of *Mycobacterium tuberculosis* was found too. This is the main sigma factor, indispensable for growth in both *Mycobacterium tuberculosis* and *Mycobacterium smegmatis*, being maintained at a constant level under various stress conditions [25]. All genes involved in iron uptake were found in both genomes. The DIP0733 protein was found in both genomes. This is a multi-functional virulence factor of *C. diphtheriae* involved in the adhesion, invasion of epithelial cells, and induction of apoptosis [26].

CRISPR-Cas is an adaptive immune system present in many bacteria and archaea, which allows cells to recognize and destroy invading genetic material, such as plasmids or phages. The CRISPR-Cas system is composed of two main parts: the CRISPR cluster (clustered regularly spaced short palindromic repeats) and the CRISPR-associated genes (cas). The CRISPR cluster contains repetitive DNA sequences interspersed by variable spaces that correspond to exogenous DNA sequences. Cas genes encode proteins that form the gene editing complex responsible for cutting invading DNA that corresponds to the variable spaces of the CRISPR cluster [27, 28]. In this study, we found Type I-E CRISPR-Cas systems in both strains. One of the sixteen spacers in each strain is known using the CRISPR-Cas + + database, matching with *C. diphtheriae* bv. mitis PC0646. Using the CRISPRTarget server, 7 spacers sequences are known. Of these, five matched with corynebacterial (*C. diphtheriae* and *C. ulcerans*). One spacer sequence matched with *Marinobacter nauticus*, a Gram-negative rod found in sea water able to degrade hydrocarbons. According to Carreira and coworkers (2018), this species is a moderate halophile and therefore could potentially be used in high saline wastewater, for which its role as a denitrifier is crucial to remove nitrate and nitrite content from industrial sources [29]. Another spacer sequence matched the *Syntrophomonas wolfei*, a Gram-negative, slightly helical rod, anaerobic, syntrophic, and fatty acid-oxidizing [30]. Of the 16 spacer sequences, half are unknown, indicating that there is an unexplored reservoir of corinebacteriophages and plasmids. *C. rouxii* strains share the same repeat consensus sequence shown in the Fig. 3.

## Conclusions

This is the first genomic study of non-toxigenic *C. rouxii* strains isolated from purulent infection in the ear and head injuries of cat in Brazil. Resistance genes and diphtheria toxin gene were not predicted in both strains, but genes involved in the regulation of diphtheria toxin, iron uptake and adherence were found. The adaptive immune system named CRISPR-Cas was found. Analyzes of spacer sequences indicate previous contact with species of the genus *Corynebacterium* and environmental species such as *Marinobacter nauticus* and *Syntrophomonas wolfei*. The genomic analyses performed in this study may help to understand the potential pathogenesis of non-toxigenic *C. rouxii* strains.

## Methods

### Origin of bacterial strains

Two *C. rouxii* strains causing head and ear injuries in a 5-year-old cat were sent to the Laboratory of Diphtheria and Corynebacteria of Clinical Relevance of the State University of Rio de Janeiro. *C. rouxii* strains 70,862 and 70,863 were deposited at the Collection of Bacteria from Environment and Health (CBAS) of Oswaldo Cruz Foundation (Fiocruz) under the numbers CBAS 829 and CBAS 830, respectively.

### Genome sequencing, assembly, and annotation

The genomic DNA extraction from the two strains was performed according to Kit Gen Elute Mammalian Mini-prep (Sigma-Aldrich). Next-generation sequencing was performed using Illumina HiSeq 2500 sequencer (Illumina Inc, USA). A library was constructed with the Nextera XT DNA Library Preparation Kit (Illumina). The reads were assembled *de novo* using the SPAdes v3.15.4 [31]. The quality control checks in FastQC software [32]. The genomes were annotated using the NCBI Prokaryotic Genome Annotation Pipeline.

### Species identification by *rps* genes and genomic taxonomy

The genomes were submitted to the rMLST database for species identification [18]. The Average Nucleotide Identity (ANI) was calculated according to the OrthoANI algorithm using the OrthoANI tool v0.93.1 [12]. DNA-DNA hybridization (DDH) was determined *in silico* for these genomes using Genome-to-Genome Distance Calculator (GGDC) v.3.0 by the BLAST method. The results were based on recommended formula 2 (identities/HSP length), the most robust for incomplete draft genomes [33]. The strains were compared with the genomes of type strains of species belonging to the *C. diphtheriae* complex.

### Determination of sequence type

For each strain, the MLST profile was determined by *in silico* extraction from WGS data using the Institut Pasteur MLST database (https://bigsdb.pasteur.fr/diphtheria/). MLST alleles of *C. rouxii* strains available in the MLST database were used to build a phylogenetic tree using MEGA version 11.0.10 [34].

### Virulence factors and antimicrobial resistance genes

The presence of resistance genes acquired by horizontal transference genes was verified by upload of genomes in the Center for Genomic Epidemiology tool ResFinder version 4 [35]. Mutations in *housekeeping* genes *gyrA* and *rpoB* were investigated from the alignment of genes in the Clustal W program [36] using *C. rouxii* FRC0190^T^ as a standard for alignment. The prediction of bacterial virulence factors was determined by the VFDB database and analyzed using VFAnalyser [37].

### CRISPR-Cas system identification

CRISPRCasFinder was applied to identify the CRISPR-Cas system of genomes. CRISPR arrays with low evidence (0 or 1) were not included in the analyses [13]. The type of CRISPR-Cas cassette was determined following the nomenclature and classification previously described [27]. Spacer sequences were analyzed for their identity using the CRISPRTarget database, and the cut-off score was the default parameters [38], and the CRISPR-Cas + + database with E-value = 0.01 [13]. Spacer hits were selected from the CRISPRTarget and CRISPR-Cas + + databases with a cut-off Identity Cover (IC) score of 0.80 [39]. The conservation of direct repeats was represented by WebLogo4 version 3.7.12 [14].

### Phylogenetic relationship

The sequences of the seven housekeeping genes (*atpA, dnaE, dnaK, fusA, leuA, odhA* and *rpoB)* of all *C. rouxii* strains deposited in Institut Pasteur MLST database (https://bigsdb.pasteur.fr/diphtheria/), plus strain type FRC 0190 (Genbank accession number LR738855), were used in the construction of the phylogenetic tree, using the program MEGA 11.0.10 [34]. The sequences were aligned using Clustal W in the Bioedit program [40].

## Data Availability

All data generated or analyzed during this study are included in this published article. The whole genome sequences of *Corynebacterium rouxii* 70862 and 70863 strains were uploaded in NCBI with accession numbers of JARUHM010000000 and JASFAY010000000, respectively.

## Abbreviations

ANI: Average Nucleotide Identity
CRISPR-Cas: Clustered Regularly Interspaced Short Palindromic Repeats
DDH: DNA-DNA hybridization
MLST: multilocus sequence typing
ST: sequence type
